# Performance evaluation of a low-cost, novel vanadium nitride xerogel (VNXG) as a platinum-free electrocatalyst for dye-sensitized solar cells†

**DOI:** 10.1039/d0ra06984a

**Published:** 2020-11-11

**Authors:** Subashini Gnanasekar, Prashant Sonar, Sagar M. Jain, Soon Kwan Jeong, Andrews Nirmala Grace

**Affiliations:** Centre for Nanotechnology Research, VIT Vellore 632014 Tamil Nadu India anirmalagladys@gmail.com; School of Chemistry and Physics, Queensland University of Technology Brisbane Queensland 4000 Australia; Centre for Material Science, Queensland University of Technology Brisbane Queensland 4000 Australia; Concentrated Solar Power Center for Renewable Energy Systems, School of Water Energy and Environment, Cranfield University Cranfield MK43 0AL UK; Climate Change Technology Research Division, Korea Institute of Energy Research Yuseong-gu Daejeon 305-343 South Korea jeongsk@kier.re.kr

## Abstract

A vanadium nitride xerogel (VNXG) was synthesised by a simple and effective method of ammonialising a vanadium pentoxide xerogel at a higher temperature. Xerogel-structured materials possess salient features such as high surface area, tunable porosity and pore size that result in enhancing the catalytic activity by a fast electron-transport pathway and increase electrolyte diffusion channels. Metal nitrides are reported as promising alternate low-cost counter electrodes to replace the conventional and expensive platinum (Pt) counter electrode. Though few studies are reported on aerogel-based CEs for DSSCs, the present work is the first attempt to synthesize and evaluate the performance of xerogel-structured metal nitrides as counter electrode materials for dye-sensitized solar cells. The synthesized material was well characterized for its structural and morphological characteristics and chemical constituents by photoelectron spectroscopy. Finally, the VNXG was tested for its electrocatalytic performance as a choice of counter electrodes for dye-sensitized solar cells (DSSCs). The photo-current studies were performed under standard 1 SUN, class AAA-simulated illumination with AM1.5G. The consolidated results revealed that the vanadium nitride xerogel exhibited good photocatalytic activity and low charge transfer resistance. This identified it as a promising low-cost counter electrode (CE) material for dye-sensitized solar cells. The photo-current conversion efficiency of the vanadium nitride xerogel CE-based DSSC reached 5.94% comparable to that of the conventional thermal decomposed Pt CE-based DSSC, 7.38% with the same iodide/triiodide electrolyte system. Moreover, the 28 days stability study of VNXG CE DSSCs provided an appreciably stable performance with 37% decrement in the PCE under the same test condition.

## Abstract 2

A vanadium nitride xerogel (VNXG) was synthesised by a simple and effective method of ammonialising a vanadium pentoxide xerogel at a higher temperature. The electrochemical and photo-current studies were performed towards a counter electrode for DSSC.

## Graphical abstract

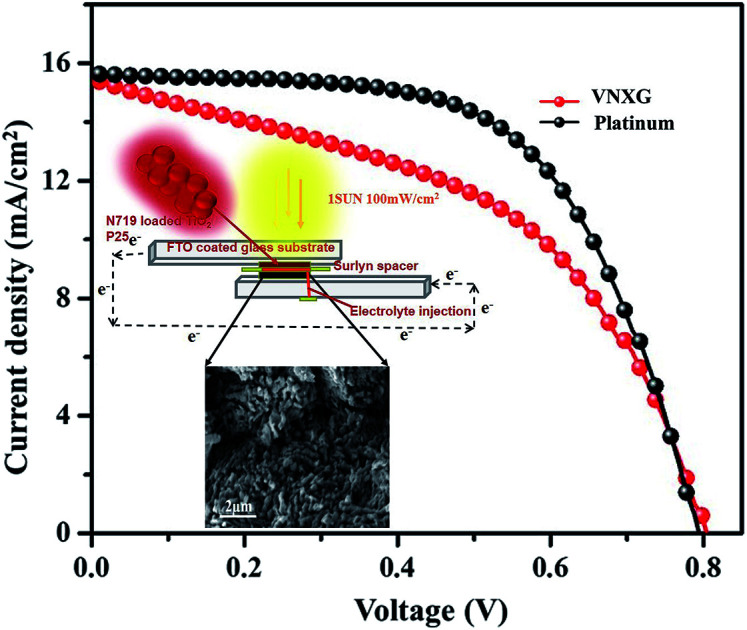

## Introduction

As per the update given by the International Renewable Energy Agency (IRENA), the world energy demand has been increased by 2.3% last year. The reported data revealed that the steep growth in the renewable energy capacity continued in 2018 with global additions of 171 gigawatts (GW). The annual increase of 7.9% was bolstered by new additions from solar and wind energies, which accounted for 84% of the growth.^1^ The rapid and massive deployment of renewable energy is important for the international community to achieve the central goal of the Paris climate change agreement, which holds the global average temperature rise close to 1.5 degrees Celsius, which is crucial to avoid the worst impacts of climate change.^2^ Among the different renewable energy resources, solar energy has been identified as an abundant, cost-efficient source of energy. Tremendous research advancements have been made in all types of solar cells: first-generation silicon-based photovoltaics, second-generation thin film-based solar cells and third-generation emerging PVs, which include dye-sensitised solar cells, perovskites, quantum dots, and organic/polymer solar cells.^3–6^ Each type of solar cell has its own pros and cons such as high efficiency but high cost, toxicity and low stability. However, dye-sensitized solar cells are one among those investigated intensively, and they have been concluded as comparatively cost-effective, efficient, stable and, most importantly, non-toxic solar cells.^7–9^ Recently, DSSCs have emerged as highly promising candidates for semitransparent and transparent windows that can generate electricity for futuristic smart cities.^10^

Dye-sensitized solar cells and their components perform exactly the same functions following the principle and operation of natural photosynthesis that occur in plants for generating energy. A typical DSSC has four components, namely, (i) a dye as the primary component for absorbing sunlight for photoelectron generation, (ii) a nanocrystalline semiconducting material that helps anchoring the dye and for the generation of electron–hole pairs (exciton), (iii) an electrocatalytic counter electrode for transmitting the generated electron–hole pairs to complete the circuit and finally (iv) an iodide/triiodide redox electrolyte for the regeneration of the dye.^11,12^ The efficient UV-visible light-absorbing dyes are used as a sunlight absorber, loaded and anchored with semiconducting oxide nanomaterials such as titanium oxide (TiO_2_) and zinc oxide (ZnO). This dye-loaded semiconducting film acted as a photoanode. Ruthenium-based dyes are found to be more efficient and conventionally used in the midst of different dyes tested to improve the absorption. Among the various redox couples tested, the triiodide/iodide (I_3_^−^/I^−^)-based redox is commonly used due to its efficient performance in DSSCs for the regeneration of dye molecules. Platinum has attained the name as an efficient counter electrode after complete theoretical and experimental validation, however the higher cost and scarcity of platinum bottleneck the commercialization of DSSCs in the economic market.^17–20^

Thus, primary research to crosscut the cost of the DSSC device is undergoing in various aspects either by reducing the usage of Pt by making composites with carbon^21,22^ or by replacing with other transition metals,^23^ metal oxides,^24^ metal nitrides,^25^ metal sulphides,^26–28^*etc.* Since the study on metal nitrides has proved the efficient catalytic activity like Pt, it is one of the significant choices for CEs, and the cost is also significantly lesser than platinum.^29,30^ At this point, intensive research has been triggered to replace platinum CEs with various transition metal nitrides such as TiN,^31,32^ Mo_2_N,^33,34^ Fe_2_N,^35,36^ NiN,^37^ W_2_N,^36^ and VN^13,14^ with diverse morphologies. Vanadium nitride is the low-cost inorganic material with high electrical conductivity and electrocatalytic activity, which is an active material in energy conversion and energy storage applications.^38–42^ A number of reports demonstrated vanadium nitride (VN) as one of the promising counter electrode reported for DSSCs.^13,15,43^ Vanadium nitride peas synthesized by a urea-metal chloride route showed high catalytic activity and reported 7.29% power conversion efficiency (PCE) compared with Pt (PCE) 7.68% for I_3_^−^/I^−^-based DSSCs.^13^ A 3D architecture composite of porous vanadium nitride nanoribbons and reduced graphene oxide showed good stability towards I_3_^−^/I^−^ redox electrolytes and reported photon-to-current conversion efficiency of about 7.43%, which is comparable to the conventional thermally decomposed Pt (7.74%).^14^ A 3D porous vanadium nitride nanoribbon aerogel was prepared by hydrothermal synthesis followed by ammonialization at high temperatures, which was used as a CE for the DSSC. This porous material enhances the electrocatalytic activity by increasing the electron transport path and resulted in its DSSC device PCE of 7.05% very close to Pt (7.43%).^16^ The record of vanadium nitride and its composite materials showed the commendable performance close to that of platinum. The reports on vanadium nitride (VN)-based counter electrodes were consolidated in Table 1. Thus far, various types of vanadium nitride nanostructures were developed with quite interesting properties for catalytic applications.

**Table 1:** Summary of performance reported on vanadium nitride based counter for DSSC

Counter electrode	Synthesise method	Electrolyte	*η* (%)	Ref.
VN peas	Urea-metal chloride route	I^−^/I_3_^−^	7.29%	13
3D porous vanadium nitride nanoribbon/reduced graphene oxide (PVNN/RGO) composite	Hydrothermal followed by thermal annealing	I^−^/I_3_^−^	7.43%	14
VN nanoparticle	High temperature sintering	I^−^/I_3_^−^	5.85%	15
Three dimensional vanadium nitride nanoribbon aerogel	Hydrothermal followed by ammonialization	I^−^/I_3_^−^	7.05%	16
VN xerogel	High temperature ammonialization of V_2_O_5_ xerogel	I^−^/I_3_^−^	5.94%	Present work

There are several routes to prepare the nanomaterials, and it can be derived in different forms such as powders, aerogels or xerogels depending on the drying process. Xerogel nanostructures are crosslinked particle networks formed by drying gels with unhindered shrinkage, which inhibits high porosity and high surface area with a controllable pore size. Xerogel-structured materials are gaining interest due to their simple preparation methods and their contributions in the composite preparation by shrinkage mechanism. By adequate tuning of their surface chemistry and textural properties, it is possible to optimise a catalyst suitable for specific applications. Few studies have been successfully reported demonstrating the enhanced performance of dye-sensitized solar cells with xerogel-structured photoanodes.^44–47^ These results encouraged the present approach to synthesize the vanadium nitride xerogel and investigate its catalytic activity towards the counter electrode for DSSCs.

Herein, a simple method was followed for the preparation of the V_2_O_5_ xerogel, and then consecutive ammonialization at high temperatures was done to prepare the vanadium nitride xerogel (VNXG). Complete evaluation of the structure and morphology of the as-prepared samples was performed. The xerogel nanostructure enhances the electrocatalytic accessibility with its interconnected hierarchical porous structure, which favours the electron transport pathway and electrolyte diffusion. This is the first attempt to synthesise the VN xerogel and test its performance towards a counter electrode for dye-sensitized solar cells. The electrochemical and *I*–*V* studies revealed the excellent characteristics of VN with this unique structure, which paved room for the optimization of the xerogel pore structure for efficient catalytic performance.

### Experimental methods

A simple and efficient V_2_O_5_ xerogel synthesis procedure followed by consecutive ammonialization at high temperatures was performed for the synthesis of the VN xerogel (VNXG). In a typical process, 2 g of vanadium pentoxide (V_2_O_5_) was mixed with 40 ml of 30% H_2_O_2_ at 0 °C. The partial decomposition of H_2_O_2_ turns the dissolved solution immediately into a clear orange solution, and the continuous evolution of oxygen further turns the solution into deep red after 3 h. The solution was stirred continuously for 6 h followed by aging for 3 days to form as a gel and subsequently dried at 100 °C for 24 h. The dried sample is V_2_O_5_ xerogel. Further, the dried material was subjected to high-temperature ammonia (160 sccm) treatment at 800 °C for 2 h for stable nitride crystallization, which was cooled to RT for further characterizations.

### Preparation of the counter electrode

The counter electrode was prepared using a doctor blade technique on a properly cleaned FTO-coated glass substrate (resistivity: 7 Ω □^−1^, Sigma Aldrich). The substrate was ultrasonically cleaned for 15 minutes using each soap solution, DI water, acetone and finally isopropyl alcohol (IPA) and dried under N_2_ flush. The coating area of the electrode was fixed to 0.4 × 0.4 cm^2^. The VNXG counter electrode coating paste was prepared using 10 mg of the as-prepared VNXG, 200 μl of DI and 20 μl Triton X. The uniform paste was coated over FTO-coated glass substrate and heated to 280 °C for 30 min to remove the binder. To validate the electrochemical performance of the VNXG, the universally accepted thermally decomposed platinum electrode was prepared and tested under the same conditions for comparison. A Pt electrode was prepared by the same doctor blade technique using 10 mM H_2_PtCl_6_ dissolved in IPA and thermally decomposed at 450 °C for 30 min in an air atmosphere.

### Preparation of photoanodes

For the preparation of the TiO_2_ electrode, first a TiO_2_ compact layer was formed on the ultrasonically cleaned FTO-coated glass substrate by treating it with 40 mM TiCl_4_ at 80 °C for 1 h, rinsed with DI water and then dried under N_2_ flow. Second, a uniform TiO_2_ paste was prepared with a commercial P25 TiO_2_ powder by subsequent mixing and grinding of 500 μl of DI water, 50 μl of 0.1 M HNO_3_ and 20 μl of Triton X. The above mixture was coated on the TiO_2_ compact layer and sintered with step-by-step temperature ramping at 350 °C for 15 min, 400 °C for 10 min and then at 450 °C for 30 min. The substrate temperature was cooled down to 80 °C and then immersed in 0.5 mM N719 dye dissolved in an ethanol solution for 24 h. The dye-loaded TiO_2_ photoanode electrode was rinsed with ethanol to remove excess dye and flushed with N_2_ and stored under dry dark conditions.

### Dye-sensitised solar cell fabrication using the VNXG and Pt counter electrode

The DSSC was assembled by sandwiching the N719-loaded TiO_2_ photoanode with the VNXG or Pt CE with thermoplastic hot-melt Surlyn as a spacer and with injected redox I_3_^−^/I^−^ electrolyte prepared with 0.05 M I_2_, 0.5 M LiI and 0.1 M 4-*tert*-butylpyridine in an absolute acetonitrile solution. The active area for the testing of the cell was fixed as 0.16 cm^2^.

### Material characterization

The crystallization and structural evaluation were performed using an X-ray diffractometer (Bruker D8) equipped with a Cu Kα radiation source (*λ* = 1.54 Å) and by Raman spectroscopy using a HORIBA spectrometer with a 532 nm laser source respectively. Field emission scanning electron microscopic (FESEM) imaging with elemental analysis (EDAX) was done using a HITACHI instrument to study the morphology and elemental composition. A high-resolution transmission electron microscope (HRTEM-FEI Tecnai G2STwin) was used to further analyse the size and shape of the particles formed. The specific surface area of the sample was measured using a BET plot with a N_2_ adsorption/desorption isotherm of the sample obtained using QUANTACHROME at 77 K. The electrocatalytic activities of the electrode were tested by cyclic voltammetry, electrochemical impedance spectroscopy and Tafel polarization analysis. All these techniques were performed using CHI instruments 660C with the active area of the coated substrate fixed as 0.16 cm^2^ for testing. Cyclic voltammetry was performed with three electrode systems: an FTO-coated CE serving as the working electrode, a Pt mesh as the counter electrode and an Ag/AgCl electrode as the reference electrode. Electrochemical impedance spectroscopy was carried out with a symmetric cell configuration at an amplitude of 10 mV under the dark condition in the frequency range from 10^5^ Hz to 0.1 Hz and Tafel polarization measurements were performed using a sandwiched symmetric CE dummy cell. The photocurrent–voltage characteristics of the assembled DSSC was tested using an Oriel Sol3A class AAA 1SUN solar simulator with a Keithley 2400 source meter under AM1.5G.

## Results and discussion

**Fig. 1:**
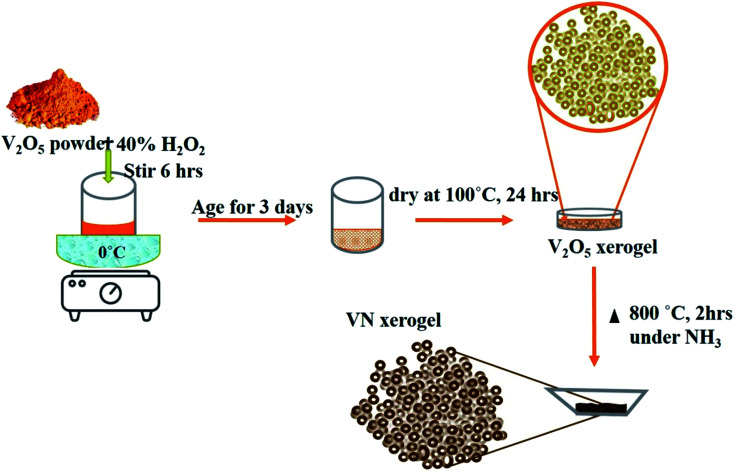
Schematic of the overall step-by-step synthesis process of the VN xerogel.

The formation mechanism of VN from the high-temperature ammonia treatment of the V_2_O_5_ xerogel and its schematics are shown in Fig. 1. In the process of V_2_O_5_ xerogel preparation, the solution of the V_2_O_5_ precursor was mixed with aqueous H_2_O_2_, leading to the formation of diperoxo anions [VO(O_2_)_2_], which is a highly exothermic reaction. The pH of the resulting orange solution is very low since H_2_O_2_ is highly acidic and pentavanadate is unstable in this solution. Then, the as-formed peroxo species is continuously oxidized in excess H_2_O_2_, which releases oxygen gas and decomposed into monoperoxo and oxo V^v^. After the completion of the reaction with H_2_O_2_, an aqueous solution of [VO_2_]^+^ and [H_2_V_10_O_28_]^4−^ was formed. Peroxo ions [O_2_]^2−^ act as chelating bidentate ligands that prevent condensation and avoid precipitation. Upon ageing and subsequent condensation of the clusters or monomers, the solution forms a homogeneous viscous dark red gel skeleton with fibril vanadium oxide, which is the V_2_O_5_·*n*H_2_O gel.^48^ The peroxo method to form V_2_O_5_·*n*H_2_O is easy and advantageous to avoid foreign ions. The concentration of vanadium can control the gel network by the addition of a volume of H_2_O_2_ to the V_2_O_5_ powder. The pseudomorphic conversion of V_2_O_5_ to VN process was performed by the high-temperature ammonia treatment of the as-prepared V_2_O_5_·*n*H_2_O gel. As the temperature increases, the process continues with the subsequent reduction of V_2_O_5_ (V_2_O_5_ → V_4_O_9_ → VO_2_ → V_2_O_3_ → VO_0.9_), and finally, the presence of ammonia hinders the topotactic substitution of nitrogen for oxygen.^49^ The overall reaction process producing VN from V_2_O_5_ and ammonia is given in eqn (1): 1
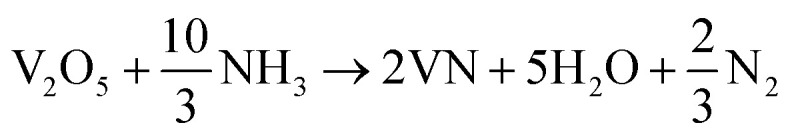


**Fig. 2:**
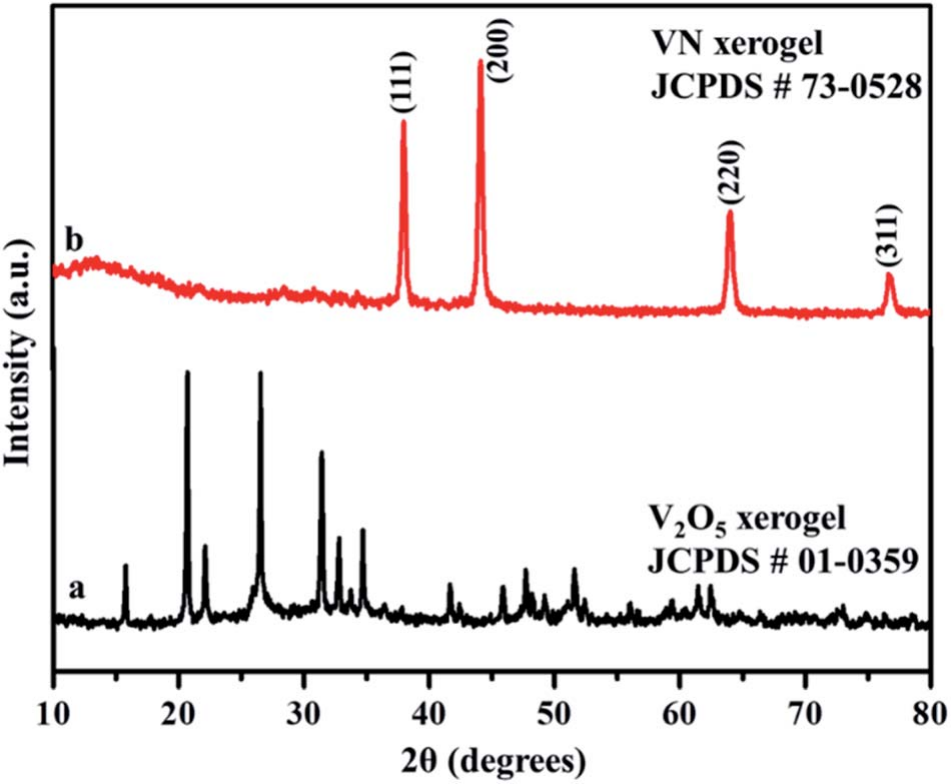
X-ray diffraction pattern of the synthesized (a) V_2_O_5_ xerogel and (b) VN xerogel.

X-ray diffraction analysis was carried out to identify the phase and purity of the synthesized samples. The XRD peak of the V_2_O_5_ xerogel and its respective ammonialized VN xerogel at 800 °C are shown in Fig. 2. All the diffraction peaks in Fig. 2a correspond to V_2_O_5_ indexed to JCPDS no. 01-0359. This V_2_O_5_ ammonialized material exhibits sharp diffraction peaks at 2*θ* values of 38.0, 44.2, 64.0 and 76.8 corresponding to the crystal planes (111), (200), (220) and (311) respectively (Fig. 2b). The peaks ascribe to the cubic crystal structure with a *Fm*3̄*m* space group and correlate well with the indexed VN (JCPDS no. 73-0528) with crystal parameters *a* = *b* = *c* = 4.13 Å and *α* = *β* = *γ* = 90°.

**Fig. 3:**
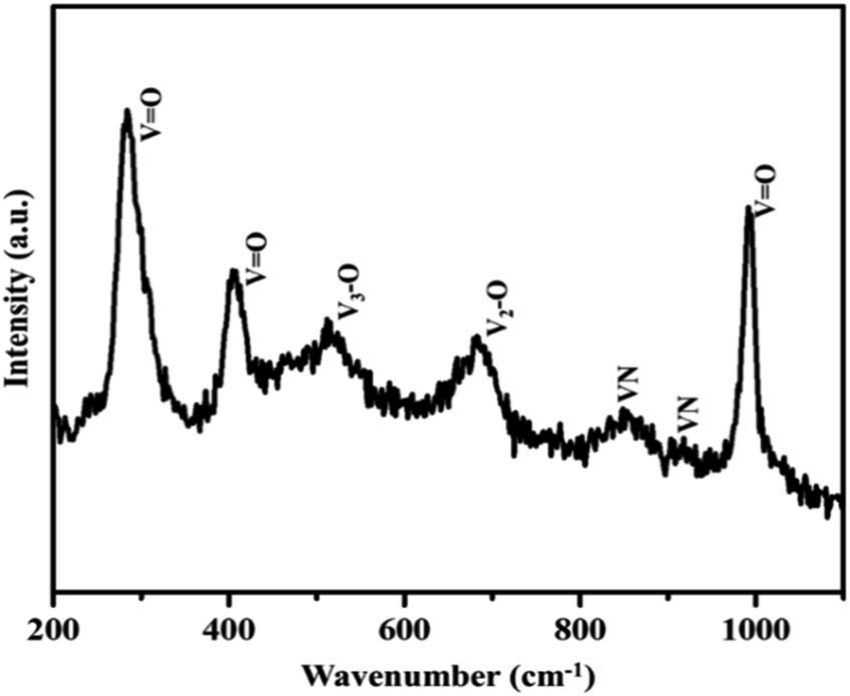
Raman spectroscopy analysis of VNXG.

**Fig. 4:**
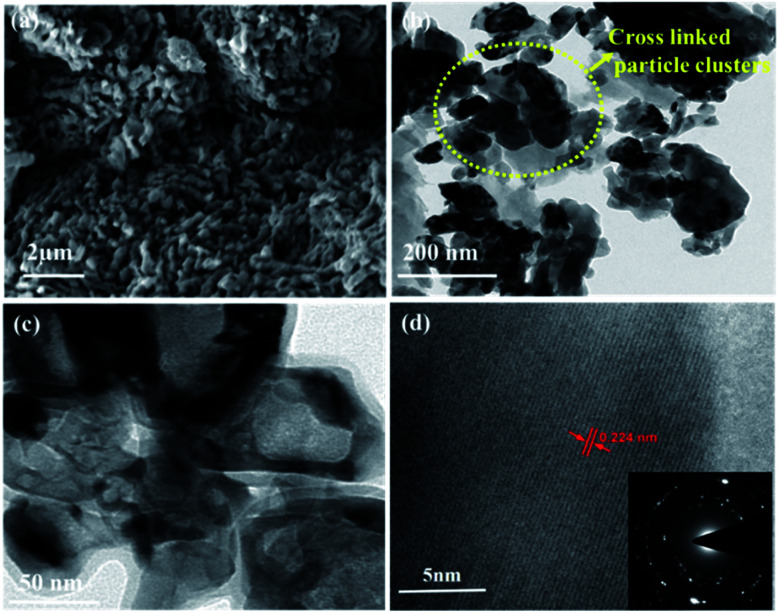
(a and b) FESEM images of the VN xerogel at different magnifications. (c) HRTEM images of the VN xerogel. (d) Diffraction images of the VN xerogel with a selected-area diffraction pattern (SAED) (inset).

Moreover, the strong and sharp peaks denote the crystallization of vanadium nitride. The XRD peak at 2*θ* around 13° shows the presence of amorphous carbon with very low peak intensity, which is from the preabsorbed contamination of hydrocarbon over the surface of VN. XPS analysis also evidenced the traces of carbon in the vanadium nitride xerogel. The structural confirmation was further performed by Raman spectroscopy with an excitation wavelength of 532 nm. Fig. 3 shows the prominent Raman peaks corresponding to the typical phases related to vanadium and oxygen at 285 (V=O), 405 (V=O), 516 (V_3_–O), 684 (V_2_–O), and 995 (V=O) cm^−1^, indicating the formation of a thin layer of vanadium oxide on the surface of VN. The low intense peak at 851 (VN) and 920 (VN) cm^−1^ are the characteristic peaks of VN. Moreover, the affinity of oxides towards vanadium is dominant, and the excitation wavelength of Raman source at 532 nm is less sensitive to interact with vanadium nitride, resulting in low-intense and scattered peaks.^43,50^ The XRD analysis of VNXG could not detect the presence of vanadium oxide due to the thin layer. The morphology of the as-prepared VN xerogel was analyzed by field emission scanning electron microscopy (FESEM), where Fig. 4a and b displays the interconnected crosslinked clusters of particles with a porous structure.

Fig. S1a and b† shows the SEM images of the commercial V_2_O_5_ powder and V_2_O_5_ xerogel, respectively. Moreover, Fig. S2† displays the cross-sectional SEM image of VN xerogel-coated FTO glass substrate with an approximate coating thickness around 30–35 μm. The high-resolution transmission electron microscopic (HRTEM) image in Fig. 4c shows the morphology of the xerogel structure with an interconnected cluster of particles with a size approximately around 50 nm. The lattice fringe spacing shown in Fig. 4d is used to calculate the fringe spacing, which was measured around 0.2 nm corresponding with the interplanar *d*-spacing of the (200) plane of VN. The inset in Fig. 4d shows the selected-area diffraction pattern of the VN xerogel representing the nanocrystalline behaviour consistent with the XRD results.

**Fig. 5:**
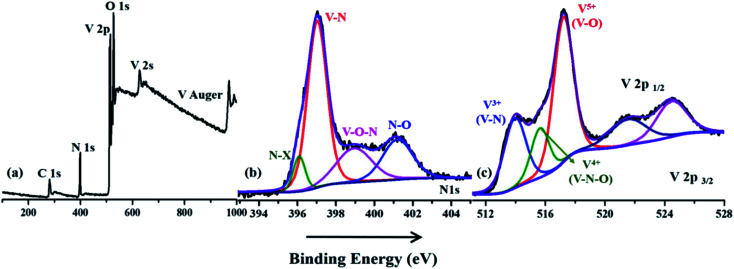
X-ray photoelectron spectroscopic image of the VNXG: (a) wide survey scan; (b) N 1s and (c) V 2p.

X-ray photoelectron spectroscopy was used to analyze the elemental composition and surface chemistry of the VNXG electrode material (Fig. 5). In Fig. 5a, the survey spectrum clearly shows the prominent presence of V 2p, N 1s, O 1s and a trace of C 1s at their corresponding binding energies. Since the surface of the vanadium has more affinity towards oxygen under the atmospheric condition, the survey shows the O 1s peaks, indicating the oxide layer on the surface of the VNXG. Moreover, the trace of carbon is from the pre-absorbed contamination of hydrocarbons over the VN surface.^51^ The high-resolution XPS spectra of V 2p_3/2_ and V 2p_1/2_ are identified with three different states V^5+^, V^4+^ and V^3+^ (Fig. 5c). The characteristic peak at binding energies 513.9 eV (V 2p_3/2_) and 521.5 eV (V 2p_1/2_) denotes vanadium in the V–N crystalline component. The peak at a binding energy of 516.2 eV shows the valance state of V^4+^, which corresponds to oxynitride V–O–N on the surface acting as a passivating layer for VN to protect from strong oxidation. Moreover, the peak at binding energies 517.2 eV (V 2p_3/2_) and 524.8 eV (V 2p_1/2_) indicates the V^5+^ valance state, the oxidation state of vanadium in the surface. In addition, the deconvolution of N 1s spectra shows the characteristic peaks at binding energies 397.0 eV, 398.9 eV and 401.2 eV corresponding to V–N, V–O–N and V–O respectively (Fig. 5b). Moreover, at a lower binding energy of 396.1 eV, the N–X peak was found, which denotes the nitrogen substitution of oxygen in the V_*y*_O_*x*_ layer.^50^ The above-mentioned XPS results of VNXG are consistent with the previous reports on VN, which supports the formation of vanadium nitride.^51–53^

**Fig. 6:**
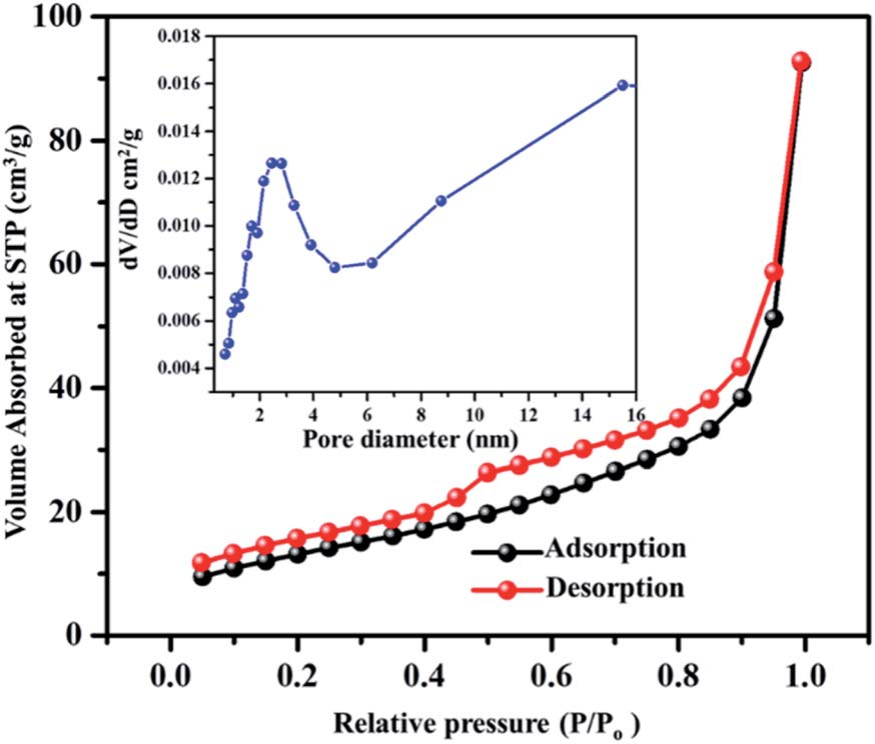
BET adsorption–desorption isotherm of the VNXG (inset: BJH pore size distribution obtained for VNXG).

The obtained VN xerogel presented interconnected crosslinked clusters of particles, which is believed to possess large surface area and excellent porosity. The porous structure and surface area of VNXG were characterized using the Brunauer–Emmett–Teller (BET) nitrogen adsorption–desorption isotherm, and the corresponding Barrett–Joyner–Halenda (BJH) pore size distribution at 77 K (Fig. 6). The obtained isotherm for the vanadium nitride xerogel was similar to the IUPAC-classified typical type IV isotherm with a H3 hysteresis loop, indicating the presence of interconnected particles and the predominant nonordered mesoporosity of the sample. Nitrogen adsorption around the relative pressure *P*/*P*_0_ (0.4–1.0) indicates a disordered lamellar pore structure with a slit and wedge-shaped pores, and the hysteresis displays the capillary condensation that occurs in mesoporous materials. The calculated BET specific surface area of VNXG was around 52 m^2^ g^−1^, and its respective pore volume was 0.368 cm^3^ g^−1^. In addition, according to the BJH pore size distribution obtained for the VN xerogel, the major pore distribution was found to be about 2–50 nm. Thus, the combination of the mesopore structure and high surface area would enhance the electrochemical active sites that improve the catalytic activity and ion transport.

**Fig. 7:**
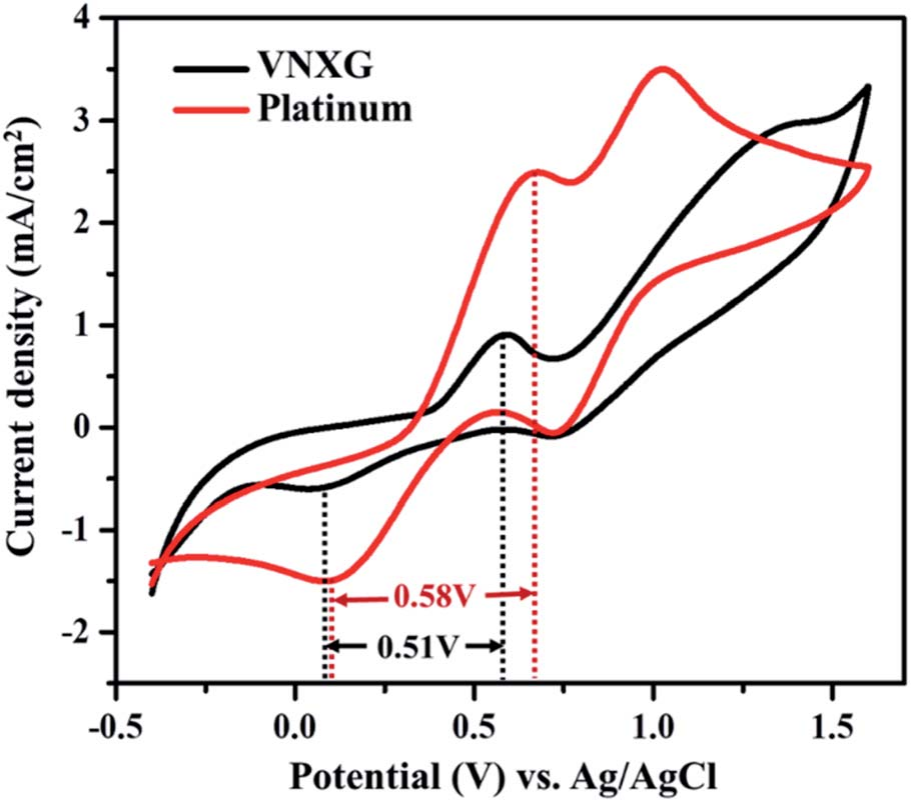
Cyclic voltammogram of the VNXG and Pt electrode at a scan rate of 50 mV s^−1^*vs.* Ag/AgCl.

The electrocatalytic activity and reaction kinetics of the CE under study were investigated by a cyclic voltammetry technique using a iodide/triiodide redox electrolyte system. The electrocatalytic activity of the CE has a direct correlation with the efficiency of the DSSC device. Fig. 7 shows the cyclic voltammetry curve of Pt and VNXG performed in a three-electrode system. An FTO substrate coated with thermally decomposed Pt or VNXG, a Pt mesh and an Ag/AgCl electrode functioned as the working, counter and reference electrodes respectively. The testing was performed with the iodide/triiodide redox electrolyte containing 0.1 M LiClO_4_, 10 mM LiI and 1 mM I_2_ in acetonitrile at a scan rate of 50 mV s^−1^ with a potential window from −0.5 to 1.8 V with reference to Ag/AgCl. Two pairs of redox peaks, reduction of I_3_^−^/I^−^ and oxidation of I_2_/I_3_^−^ were observed at low-potential and high-potential domains, which are clearly explained by eqn (2) and (3) respectively.

The redox peaks at a lower potential are attributed to the following reaction:

2 I_3_^−^ + 2e^−^ ↔ 3I^−^

The redox peaks at a higher potential are attributed to the following reaction:

3 3I_2_ + 2e^−^ ↔ 2I_3_^−^

The reduction peak corresponding to eqn (2) depicts the integral catalytic ability of CE to reduce triiodide to iodide, which is denoted as a cathodic peak current density.^54^ The reduction capacity of the CE was estimated by two characteristic parameters such as peak current density and peak-to-peak separation (*E*_pp_) of the negative redox pair, which reveals the overall electrocatalytic ability of the counter electrode. The higher the peak current density, the lower the *E*_pp_ value of the CE, which is attributed to the high catalytic performance of the electrode material.^55–57^ As observed in Fig. 7, the VNXG shows good catalytic activity by representing two pairs of redox peaks with an apparent current density similar to that of the Pt electrode. The peak-to-peak separation is calculated from eqn (4) as follows:

4 *E*_pp_ = |*E*_p_(anodic) − *E*_p_(cathodic)|

**Fig. 8:**
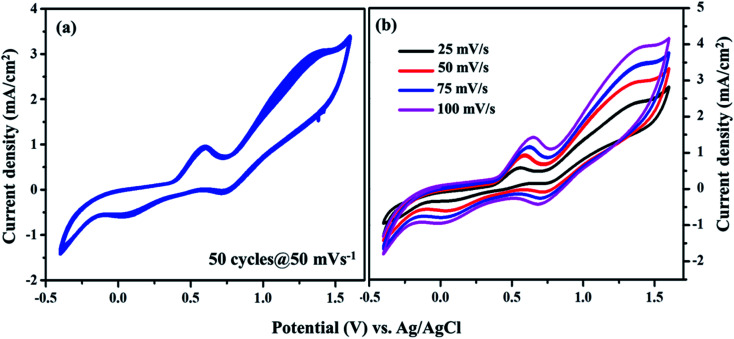
(a) Cyclic voltammogram of the VNXG tested for the 50-cycle stability study at a scan rate of 50 mV s^−1^ and (b) CV at different scan rates *vs.* Ag/AgCl.

From Fig. 7, the peak-to-peak separation (*E*_pp_) for Pt was observed as 0.58 V and for VNXG as 0.51 V. The *E*_pp_ value of VNXG is slightly lower than that of Pt, indicating higher reversibility of I_3_^−^/I^−^ on the VNXG than on Pt. Thus, the CV results in the significant peak current and low peak-to-peak separation at a lower potential reveal the favourable electrocatalytic activity of the VNXG comparable to Pt, which is attributed to the mesoporous structure of the VN xerogel that provides more active sites for the catalytic reaction and better contact with the electrolyte. Next to the electrochemical activity, the electrochemical stability is an important criterion for the material of choice as the CE for DSSCs. The electrochemical stability of the VNXG was investigated using 50-cycle successive CV scanning at a scan rate of 50 mV s^−1^ in a potential window of −0.5 to 1.8 V *vs.* Ag/AgCl (Fig. 8a). The unchanged shape and current density indicates the excellent electrochemical stability of VNXG electrode in I_3_^−^/I^−^ electrolyte system. In addition, Fig. 8b shows the CV curve of the VNXG with the I_3_^−^/I^−^ redox reaction at different scan rates from 25 mV s^−1^ to 100 mV s^−1^ to analyse the charge-transfer mechanism of the electrode. The increase in peak current density with the increase in scan rate indicates the activity increase of inner sites of the nitride electrode and the redox reaction on the surface of the CE by diffusion of the I_3_^−^/I^−^ redox pair.

**Fig. 9:**
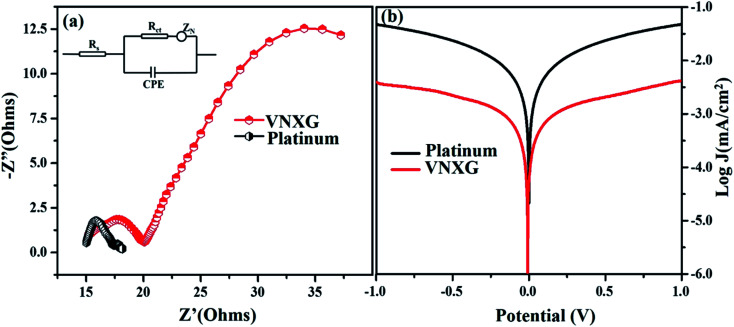
(a) Nyquist plot of electrochemical impedance spectroscopy for the VNXG and Pt CE in a symmetric cell configuration consisting of two identical CEs at 0 V under dark conditions (Inset: equivalent circuit fitted for the data using the EC-lab software). (b) Tafel polarization plot of the VNXG CE and Pt CE symmetric cell.

The intrinsic charge transfer process and transport kinetics at the CE/electrolyte interface was investigated using electrochemical impedance spectroscopy (EIS) and Tafel polarization measurements using two identical counter electrodes with a CE/electrolyte/CE symmetric cell assembly. Fig. 9a shows the Nyquist plot of the VNXG and conventional Pt with an amplitude of 10 mV under dark conditions in the frequency range of 10^5^ Hz to 0.1 Hz. The equivalent circuit inserted in Fig. 9a was used to fit the Nyquist plot using the EC-lab software, and the fitted Randles-type circuit components are summarized in Table 2. The component *R*_s_ is the ohmic series resistance, which includes bulk resistance of CE, resistance of the FTO substrate and the contact resistance obtained from the real axis intercept in the high-frequency region. The high-frequency semicircle corresponds to the contribution of *R*_ct_, the charge transfer resistance on the electrode/electrolyte interface and CPE, the constant phase element describing the capacitance of the CE/electrolyte interface, which is developed due to the accumulation of ions at the electrode surface. The low-frequency semicircle reflects *Z*_w_, the Nernst diffusion impedance of the I_3_^−^/I^−^ redox couple in the electrolyte.^58,59^ The charge transfer resistance *R*_ct_ for the VNXG is slightly higher than that of *R*_ct_ of Pt, which promises its significant performance in the solar cell device. *R*_ct_ is directly accountable to the fill factor (FF) of the DSSC. These results are in accordance with the cyclic voltammetry results as the VNXG has appreciable catalytic activity for the reduction of I_3_^−^/I^−^ and low charge transfer resistance at the electrode–electrolyte interface, which validates the favourable good photoconversion efficiency of the DSSC device with the VNXG CE comparable to the Pt CE.

**Table 2:** Summary of EIS parameters of the VNXG and Pt symmetric cell assembly and photovoltaic parameters of the DSSC assembled with the VNXG CE and Pt CE

Electrode	*R*_s_(Ω)	*R*_ct_ (Ω)	*V*_oc_ (V)	*J*_sc_ (mA cm^−2^)	FF	*η* (%)
VNXG	15.15	4.62	0.794	15.04	47.8	5.94
Platinum	15.01	2.26	0.802	15.83	59.9	7.38

The Tafel polarization analysis was performed to further understand the interfacial charge transfer resistance at the surface of the CE/redox–electrolyte interface. Fig. 9b shows the Tafel polarization curve that demonstrates the logarithmic current density *vs.* potential obtained for the symmetric cell with the VNXG and Pt CE. The polarization plot has three distinct potential zone boundaries, namely, polarization zone |*V*| ≤ 120, Tafel zone and diffusion zone |*V*| < 400. From the Tafel curve, the exchange current density *J*_0_ and the limiting current density *J*_lim_ can be obtained, which correlates the catalytic activity of the catalyst electrode. The exchange current density *J*_0_ can be measured from the Tafel zone as the tangent slope of cathodic or anodic curves with the equilibrium potential.^60,61^ It was found that the *J*_0_ value for the VNXG is lower than that of Pt, which justifies the results observed from the CV and EIS.

The limiting diffusion current density *J*_lim_ depends on the diffusion coefficient, which results in the diffusion velocity of the redox couple in the electrolyte. As shown in Fig. 9b, in the diffusion zone of the VNXG and Pt CE, the VNXG shows slightly lower values of *J*_lim_ than Pt, which shows its reasonable rate of diffusion of I_3_^−^ reduction in the electrolyte. The overall electrocatalytic study revealed that the low-cost VNXG electrode demonstrates good electrocatalytic activity towards the iodide/triiodide redox electrolyte and low charge transfer resistance, which is suitable to test it as a counter electrode for the DSSC.

**Fig. 10:**
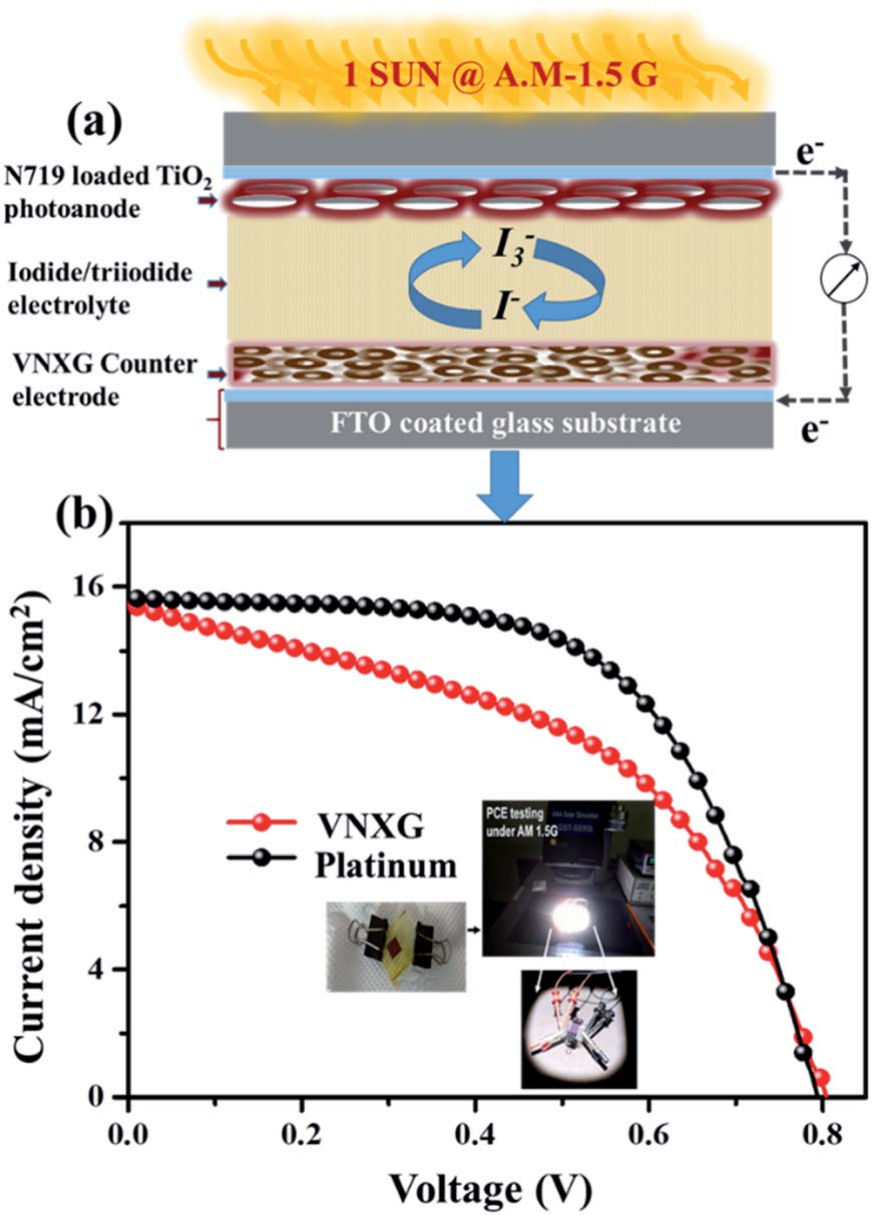
(a) Schematics of the assembled DSSC. (b) The characteristic DSSC *J*–*V* photocurrent voltage curves for VNXG and Pt CE.

The schematic and the investigation of the photovoltaic performance of DSSCs with the VNXG CE and the Pt CE are shown in Fig. 10a and b respectively. The photocurrent density–voltage (*J*–*V*) curve displays the photovoltaic parameters such as open circuit voltage (*V*_oc_) and short circuit current (*J*_sc_). The fill factor (FF) and the subsequent light-to-power conversion efficiency were calculated according to eqn (5) and (6) as follows:5
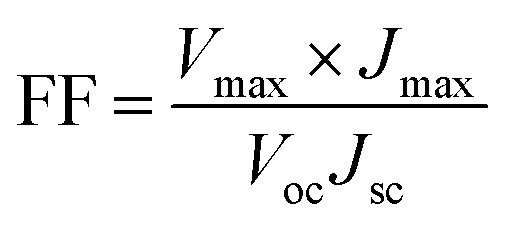
6
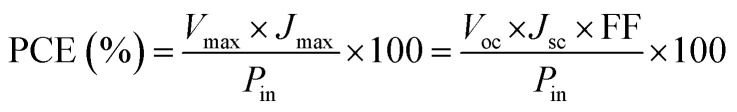
where *V*_max_ and *J*_max_ are the voltage and current density under maximum power output in the *J*–*V* curve and *P*_in_ is the power of the incident light.^62^ The photovoltaic parameters obtained according to the device performance are summarized in Table 2. The power conversion efficiency for the VNXG CE-based DSSC is 5.94% with 15.04 mA cm^−2^ (*J*_sc_), 0.794 V (*V*_oc_), and 47.8 (FF), which are comparable to the efficiency of the conventional thermal decomposed Pt CE-based DSSC, namely, 7.38% with 15.83 mA cm^−2^ (*J*_sc_), 0.802 V (*V*_oc_), and 59.9 (FF). The surface area of the electrode plays a significant role in the electrocatalytic performance of the electrode. The more and sufficient active sites promote the electrocatalytic reaction and enhance the electrocatalytic capability of the counter electrode. Though the *J*_sc_ and *V*_oc_ values of VNXG are high and comparable to Pt, the overall efficiency is slightly lower than Pt. This reasoned from the surface property and non-uniform pore distribution in the as-prepared xerogel. Appropriate tuning of the surface area and porosity can further enhance the catalytic activity and increase the performance efficiency of the DSSC device.

**Fig. 11:**
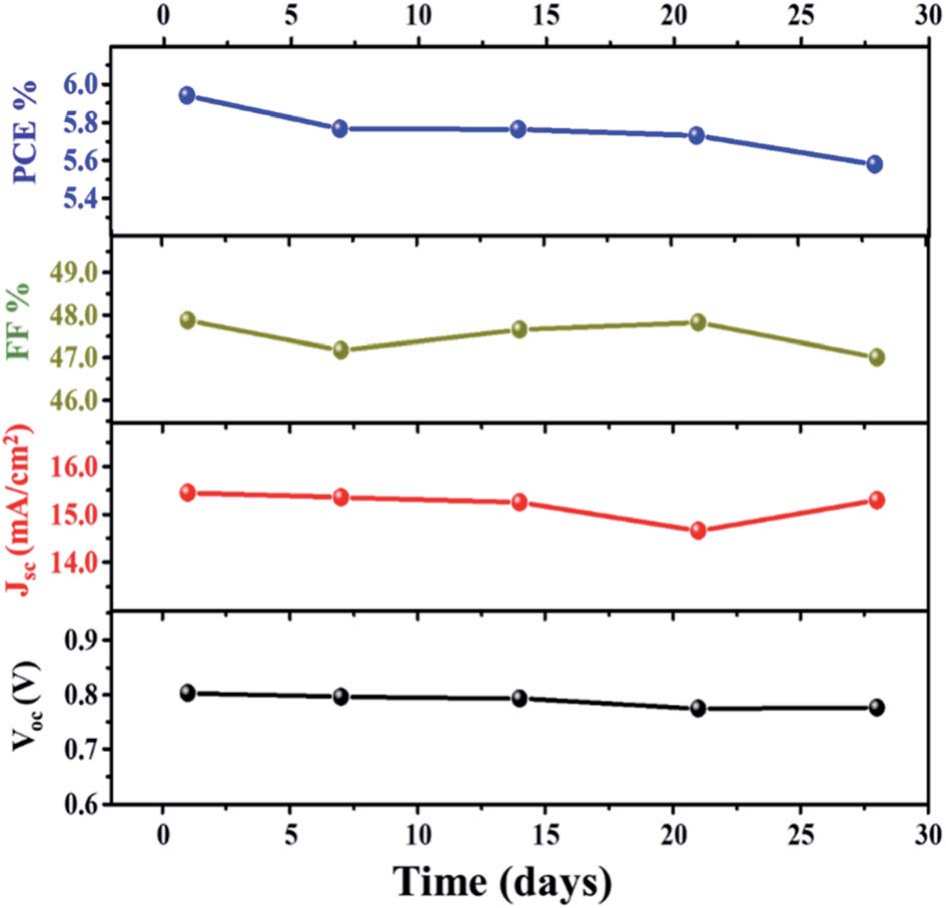
Stability study of the DSSC with the VNXG CE for 28 days.

Further investigation of the stability of the assembled DSSC with the VNXG CE was systematically examined for 28 days. After each IV measurement, the DSSC was preserved under a dark condition, and the photovoltaic performance at a subsequent interval of time was tested under standard conditions. The photovoltaic parameters obtained for the studied time frame are shown in Fig. 11. The DSSC with the VNXG tested at the 28th day shows a conversion efficiency of 5.57% with 15.29 mA cm^−2^ (*J*_sc_), 0.776 V (*V*_oc_), and 47 (FF). The result indicated about 37% decrement in the efficiency after 28 days of testing, which is an appreciably good performance under laboratory conditions, and this result promises the efficient and stable performance of the VNXG electrode as the catalyst counter electrode for DSSCs.

## Conclusion

For the first time, a VN xerogel has been successfully synthesized from a hydrothermally prepared V_2_O_5_ xerogel followed by high-temperature ammonialization, and it was systematically characterized to analyse the structural, morphological, optoelectronic and spectroscopic informations and the electrocatalytic activity towards a suitable alternative counter electrode for dye-sensitized solar cells. It is observed from the cyclic voltammetry, Nyquist plot and Tafel polarization techniques performed on the VNXG as an electrode for DSSCs that the VN xerogel shows good electrocatalytic activity and charge transfer and diffusion properties. This results in excellent performance efficiency in comparison to DSSCs made with the conventional Pt electrode. In addition, the 28 days stability test results also showed considerable good performance with 37% decrement in the PCE. The overall result promises and encourages extensive research on low-cost xerogel-structured vanadium nitride-based DSSC devices as effective and efficient alternative counter electrodes for DSSCs.

## Conflicts of interest

There are no conflicts to declare.

## Supplementary Material

RA-010-D0RA06984A-s001
