# Supplementary material for: Performance evaluation of a low-cost, novel vanadium nitride xerogel (VNXG) as a platinum-free electrocatalyst for dye-sensitized solar cells
Source: RSC Adv. 2020 Nov 11;10(67):41177–86. doi: 10.1039/d0ra06984a (PMC9057778; doi:10.1039/d0ra06984a)
Supplement: RA-010-D0RA06984A-s001 [file RA-010-D0RA06984A-s001.pdf]

Electronic Supplementary Material (ESI) for RSC Advances.
This journal is © The Royal Society of Chemistry 2020

Supplementary Material

Performance Evaluation of Low-Cost, Novel Vanadium Nitride Xerogel (VNXG) as a Platinum Free Electrocatalyst for Dye-sensitized Solar Cells

Subashini Gnanasekar,[a] Prashant Sonar,[b,c] Sagar M. Jain,[d] Soon Kwan Jeong,[e*] Andrews Nirmala Grace[a*]

[a]Centre for Nanotechnology for Research, VIT, Vellore 632014, Tamil Nadu, India.

[b]School of Chemistry and Physics, Queensland University of Technology, Brisbane, Queensland 4000, Australia

[c]Centre for Material Science, Queensland University of Technology, Brisbane, Queensland 4000, Australia

[d] Concentrated Solar Power Center for Renewable Energy Systems, School of Water Energy and Environment, Cranfield University, Cranfield MK43 0AL, UK

[e] Climate Change Technology Research Division, Korea Institute of Energy Research, Yuseong-gu, Daejeon 305-343, South Korea

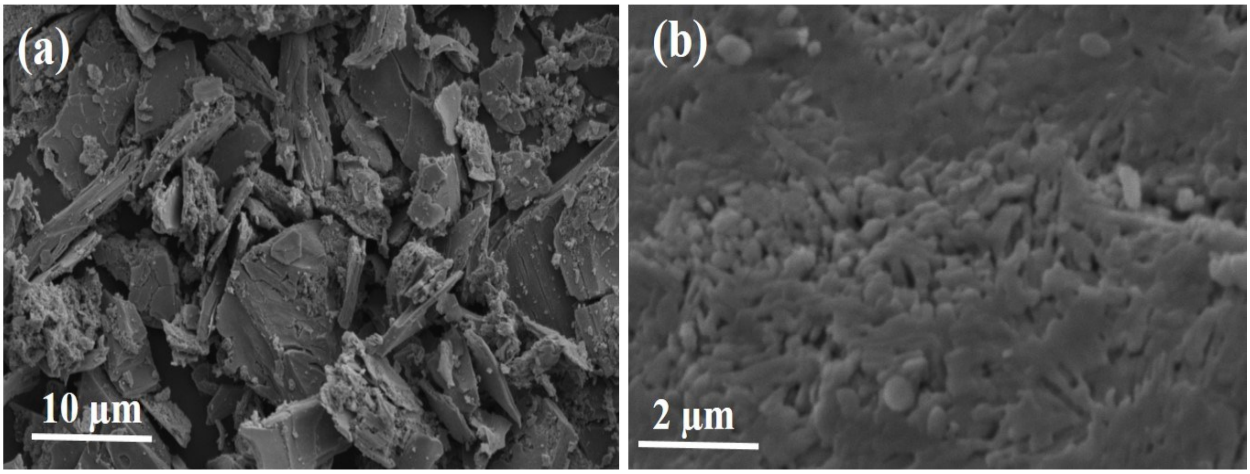

Fig. S1. SEM images of (a) commercial V_2O_5 powder (b) V_2O_5 Xerogel.

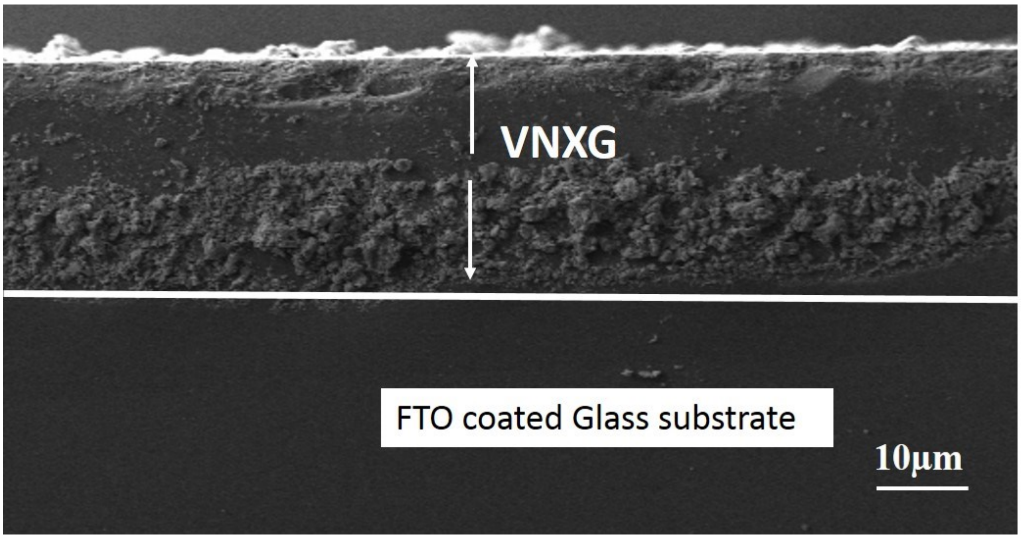

Fig. S2. Cross sectional SEM image of VN xerogel coated FTO glass substrate.
